# On the Application of Image Processing Methods for Bubble Recognition to the Study of Subcooled Flow Boiling of Water in Rectangular Channels

**DOI:** 10.3390/s17061448

**Published:** 2017-06-20

**Authors:** Concepción Paz, Marcos Conde, Jacobo Porteiro, Miguel Concheiro

**Affiliations:** School of Industrial Engineering, University of Vigo, Lagoas-Marcosende, Vigo 36310, Spain; mfontenla@uvigo.es (M.C.); porteiro@uvigo.es (J.P.); mconcheiro@uvigo.es (M.C.)

**Keywords:** machine vision, flow visualization, bubble recognition, bubble tracking, subcooled flow boiling

## Abstract

This work introduces the use of machine vision in the massive bubble recognition process, which supports the validation of boiling models involving bubble dynamics, as well as nucleation frequency, active site density and size of the bubbles. The two algorithms presented are meant to be run employing quite standard images of the bubbling process, recorded in general-purpose boiling facilities. The recognition routines are easily adaptable to other facilities if a minimum number of precautions are taken in the setup and in the treatment of the information. Both the side and front projections of subcooled flow-boiling phenomenon over a plain plate are covered. Once all of the intended bubbles have been located in space and time, the proper post-process of the recorded data become capable of tracking each of the recognized bubbles, sketching their trajectories and size evolution, locating the nucleation sites, computing their diameters, and so on. After validating the algorithm’s output against the human eye and data from other researchers, machine vision systems have been demonstrated to be a very valuable option to successfully perform the recognition process, even though the optical analysis of bubbles has not been set as the main goal of the experimental facility.

## 1. Introduction

Nowadays the compactness and lightness of heat exchanger systems are some of the keys to success in almost all industries, such as the electronic and the automotive sector. In order to achieve these goals, it is mandatory to exchange heat using latent heat transfer mechanisms even at a local scale in the traditional single-phase devices. Therefore, the better the knowledge of the boiling process, the better the design that can be achieved. This work discusses the application of machine vision (MV) systems to the characterization and tracking of the bubbles under subcooled flow boiling of water over a heated metal sheet. MV is relatively widespread and documented in many industrial processes, mainly in quality control and final checking, having been revealed to be valid for edge detection and pattern or optical characteristic recognition [1,2].

The application of photographic support to the better knowledge of the boiling process has been commonly used since the 1950s [3,4]. In the following decades, researchers studied the main aspects of nucleated boiling bubble size-time evolution [5,6,7,8], the nucleation mechanism [9,10,11], critical heat flux (CHF) [12,13,14] and studies about frequencies, diameters and trajectories [15,16,17] according to the technology of those years and, for some of them, with very interesting solutions, simultaneously employing more than one visualization method [18,19]. With the advances in high-speed photography, researchers commenced to use snapshots to formulate and validate models for different aspects of bubbling parameters, such as bubble dynamics and time evolution [20,21,22,23,24,25] and CHF and pattern analysis [26,27,28,29], as well as set down the rules for the first wall heat partitioning models. These kinds of models, which separate the different mechanisms under a physical approach, have become some of the most ambitious ways of modelling the boiling process since the end of the past century. Among the various mechanisms taking place in the heat exchange process, the main three, namely evaporation, bubble agitation and quenching, require comprehensive knowledge of the bubble parameters for the model to succeed. Therefore, several secondary modelling processes are required to get accurate information about the bubble characteristic diameter, number of active sites present and bubble releasing frequency [30,31,32]. At the beginning, the manual process was slow and tedious, but current technology brings the opportunity for turning the bubble recognition into a mass and automatized process. In the last few years, many research papers have tackled bubble size measurement and tracking procedures by the use of high speed filming as a valuable aid even with some other techniques, such as the infrared thermometry [31,33,34,35,36,37,38,39], ultrasound technologies [40] or particle image velocimetry [41,42]. However, only a few authors briefly explain the processing image steps [43,44,45,46], and almost none fully described the computational steps of the recognition process. These works generally focused on the boiling phenomena being investigated rather than on the specific methodology employed for bubble recognition and characterization. Moreover, not only the experimental equipment, but also the procedure and design of experiments employed for bubble detection may have a strong impact on the results concerning the measured bubble parameters as has been demonstrated by Yoo et al. [47,48], and thus, it should be carefully described to allow the comparison between different works. In this paper, two algorithms are presented for both the front and side projections that significantly help in the characterization of bubbles generated by a subcooled flow boiling. This computer approach allows a huge enlargement in the size of the sample of recording frames, as well as the treatment of the heated surface as a whole, giving a global view about the interactions between nucleation sites. The front projection algorithm has been previously employed in [49,50] to determine the bubble diameters and nucleation site density over different surfaces; but it has not been explained, and only some results were shown. In these works, the covered range for the different flow properties were: from 97–872 kg/s·m^2^ for mass flux, from 76.5–93.5 °C for bulk temperature and operating pressures from 110–190 kPa. The boiling process has been maintained on the nucleate regimen varying heat flux until 0.65 MW/m^2^. Above this value, image processing data were not reliable, since the system goes beyond the net vapor generation point, and bubble merging dominates the process.

## 2. Materials and Methods

### 2.1. Experimental Setup

The herein described algorithms can be easily adapted to any boiling footage, if a minimum coherence is kept in terms of the recognition pattern, as will be explained afterwards. The description presented here focuses on the illumination system and the high-speed video recording. Further details about other elements of the experimental boiling facility with no direct relationship to the MV system can be consulted in the previous work by the authors [49,51].

**Figure 1:**
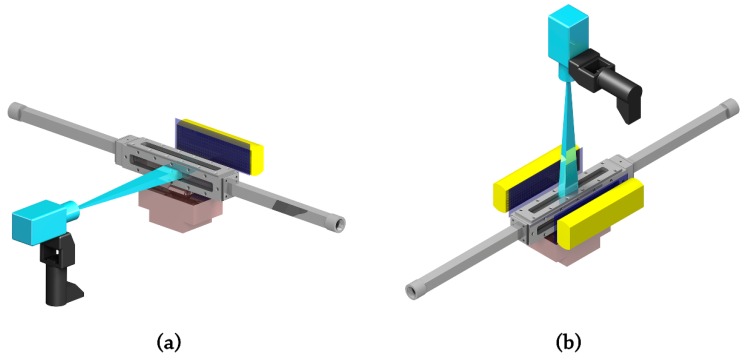
High-speed camera and lamp position: (**a**) side projection; (**b**) frontal projection.

The optical recognition of the bubbles is inevitably related with the lighting-up system. Two different illumination setups have been performed in order to record both the front and side projections. For the front projection, the arrangement consists of two lamps lighting both sides of the heated surface (Figure 1b), with the aim of characterizing the bubbles by two diametrically-opposed bright areas. In the case of the lateral or side projection, the system comprises only one lamp backlighting the scene (Figure 1a), to emphasize the contrast of the bubbles over the background. Since high-speed filming is highly demanding in terms of illuminance, each one of the dimmable LED panels used achieves a maximum luminous flux of 7700 lumen with a total consumption of 80 W. For the presented experimental setup, this leads to an illuminance of at least 600,000 lux measured at the region of interest for a single lamp.

The operating frame ratio of the camera used can be set up to a maximum of 10,000 fps, bearing in mind that the greater the recording ratio, the less the resolution. Other characteristics of the machine vision system are shown in Table 1. Since the width of the heated plate is 10 mm, the sensor resolution and the speed ratio have been adjusted to 400 × 170 pixel and 8000 fps respectively for most of the experiments with the front projection. This adjustment, together with the used lens (50 mm of focal length), allows a recording area of 23.5 × 10 mm, covering the total width of the heating section. For the side projection, higher resolutions have been used to improve the quality of the images at the expense of covering smaller areas. In general, due to the different kind of information desired in each of the situations, a balance between time resolution, spatial resolution and area covered has to be achieved. For instance, sacrificing the pixel/mm resolution to cover a wide area could be a solution when calculating site densities at very low values for the heat flux, when only a few bubbles appear on the heated plate and the density is easily influenced. By contrast, where the aim is to size and sketch the border of the bubble, the resolution should be as best as possible, forcing the reduction of the size of the region of interest. Analogous reasoning can be stated for determining the bubble nucleation frequency, where higher frame rates lead to poorer pixel/mm resolutions.

The camera owns a low latency memory module to successfully write the images, and it is connected to the computer by gigabit Ethernet for file transference, setup and monitoring purposes.

### 2.2. Machine Vision Algorithms

The two solutions presented here cover both the front and the side views of the heating surface. In the case of the frontal projection, the proposed algorithm has been able to identify each bubble and determine its nucleation site, size evolution and releasing frequency by means of a subsequent post-process of the recognized information. The obtained information would become very useful in the formulation and validation of heat flux partitioning boiling models, since they require accurate information of bubble diameter and nucleation site densities and frequencies. The solution for the side projection is focused on the bubble trajectories and size-time evolution, being useful to validate models for bubble dynamics and predictions about the boundary layer morphology. Concerning the precision of the recognition method, it will depend on the lens and camera characteristics, the filming area covered, the frame rate and the user input values for threshold and discarding criteria, so no general values can be given.

#### 2.2.1. Front Projection

This projection gives information about the bubbles’ instantaneous location on the front plane and the bubble characteristic front dimension. In order to extract any information about the nucleation sites’ location, the temporal bubble size evolution and the release frequency of the bubbles, a post-processing algorithm needed to be executed after the optical recognition. Mainly, this post-process consists of carrying out a tracking operation for each of the bubbles detected.

**Figure 2:**
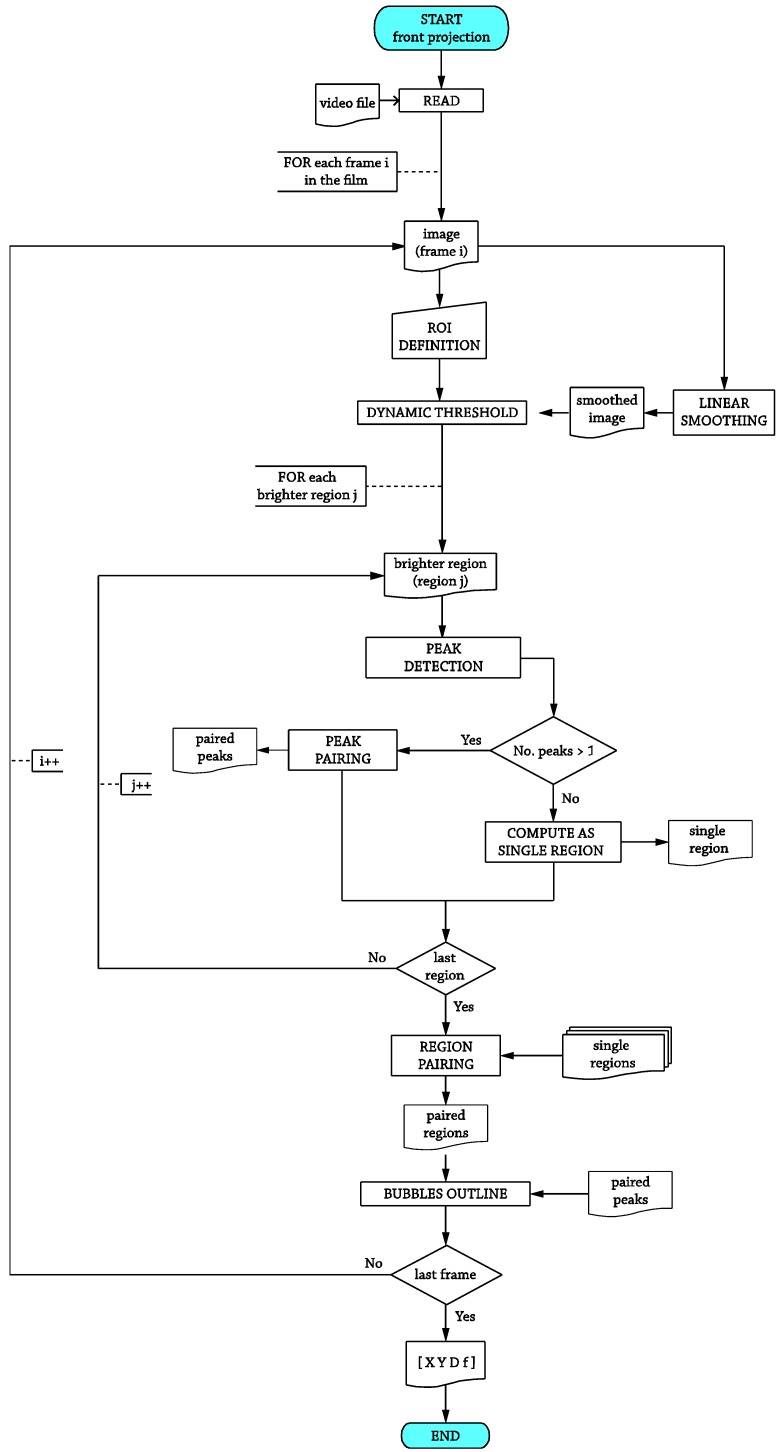
Flowchart for the front projection optical recognition.

To carry out the optical recognition, the twin lamp arrangement used here allows the characterization of the bubbles by two antipodal shiny areas. Therefore, the optical method is focused on locating those areas. The core structure for the operations performed over the media acquired using the front projection is summarized in the flow chart sketched in Figure 2.

**Figure 3:**
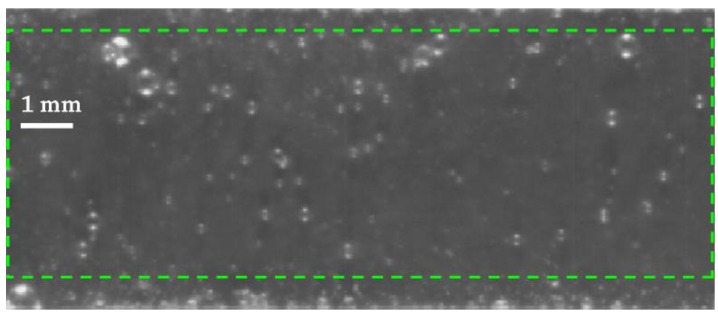
ROI definition.

The main operations to accomplish with the MV software are as follows:

● ROI definition:

This is the prior step to select the area of interest of the image (Figure 3) in which the analysis will be carried out. It can be an automatic or a manual process and permits selecting the desired areas of the image, where the recognition process is going to be done.

**Figure 4:**
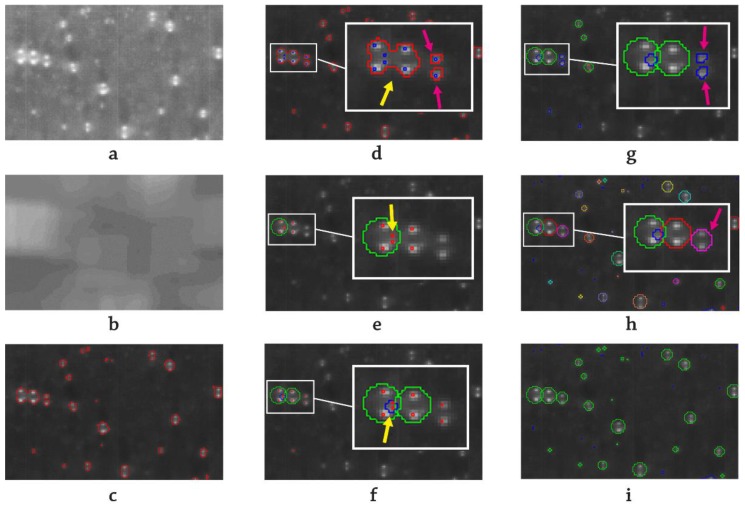
Illustrative image sequence for the front projection optical recognition T_b_ = 85 °C, p = 150 kPa, G = 484 kg/s·m^2^, q_w_ = 500kW/m^2^. (**a**) Original image; (**b**) reference image (smoothed); (**c**) bubble candidate regions; (**d**–**h**) peak pairing process; (**i**) final result (green: definitive bubbles; blue: residual areas). Images a and b have been adjusted in terms of brightness and contrast for visualization purposes.

● Dynamic threshold:

This is the first segmentation process to be made and splits the image into two regions: bubble candidate regions (red outlined regions in Figure 4c) and the rest of the image. Therefore, it is the most critical step because any valuable information not included in the useful region will be omitted in the subsequent steps. In order to improve the quality of the segmentation, a dynamic threshold operation has to be performed avoiding simple or static threshold operations in any case. There are several aspects, direct or indirect lighting flickering, changes in indirect lighting intensity, instantaneous bubble shadows and reflections, that can modify the grey threshold’s absolute value. Since these problems affect both global and local areas of the image and change with time, the operation has to be performed temporarily (frame by frame) and also spatially.

This threshold operation has two inputs, threshold value and reference image, and consists of comparing the original image (Figure 4a) with the reference one (Figure 4b) pixel by pixel. All of the pixels with a higher grey level than the grey level of the same pixel in the reference image value plus the input threshold will be marked as a bubble candidate pixel. The reference image must be representative of the medium level of grey in each local area of the image, so a global homogeneous image based, for instance, on the original image’s histogram is not recommendable. Therefore, a locally-smoothed image should be used instead. The smoothing method can be any common filter such as the Gaussian blur or the linear smoothing, as used here.

**Figure 5:**
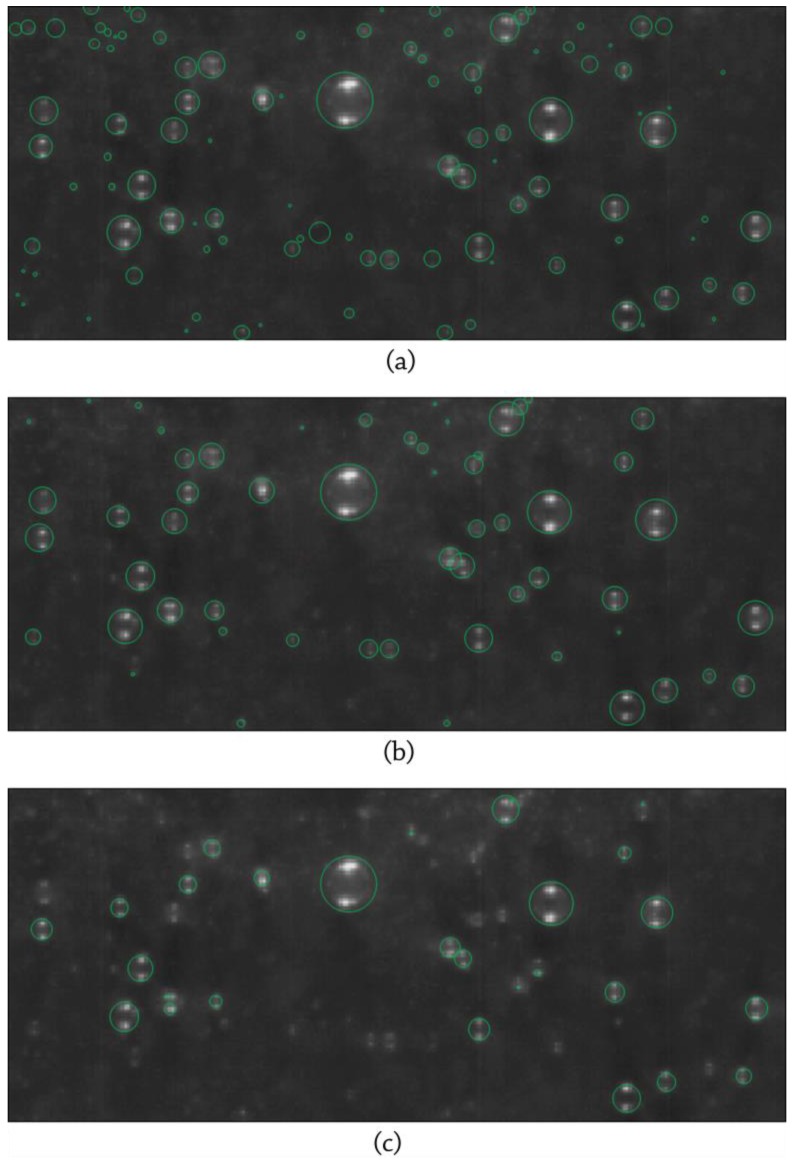
Dynamic threshold sensitivity for the front projection T_b_ = 85 °C, p = 150 kPa, G = 484 kg/s·m^2^, q_w_ = 450 kW/m^2^: (**a**) threshold value: 8; (**b**) threshold value: 16; (**c**) threshold value: 48. Absolute values on a scale from 0 (black) to 255 (white).

The threshold value has to be manually adjusted before proceeding with the batch recognition. If the value is too low, the MV output will outline a high amount of small bubbles (Figure 5a). On the contrary, if the threshold value is set to a high value, too many bubbles will be discarded (Figure 5c). The optimum selection should be a medium value, low enough to retain all of the generated bubbles during most frames in which they exit (Figure 5b). To successfully perform the bubble tracking during the post-processing phase and provided the fact the brightness of bubbles could diminish at an early stage or at the end of the bubble life, the result of a low pass threshold operation with respect to the main one is also stored for tracking purposes, as will be explained later.

● Peak detection:

This operation is the second segmentation process to be performed inside the bubble candidate regions. It consist of detecting the brightest peak (small blue squares in Figure 4d) inside a region, that is a pixel brighter than its neighbors. For each of these regions, there are two possibilities, single peak regions or multiple peak regions. In the former case (magenta arrow pointed regions in Figure 4d), the region will be marked as a single region, whereas for the latter (region pointed to with a yellow arrow in Figure 4d), a peak pairing process will take place.

● Peak pairing:

This process will identify the paired peaks by matching each pair of peaks that meets a verticality criterion, which assumes a user input tolerance. Since they belong to the same region, no other criterion should be necessary.

**Figure 6:**
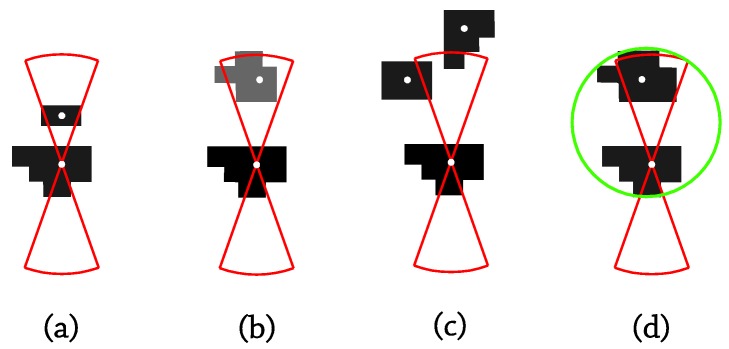
Discarding criteria in front projection. The double red cone, whose angle and height depend on the size of the selected region, indicates the valid area for the twin bright region centroid. Regions in (**a**–**c**), remain unpaired, as they do not fulfil all of the criteria.

● Region pairing:

This step is similar to the peak pairing process, but a verticality criterion alone is not enough to identify the matching regions successfully. Therefore, additional criteria have been taken into account. The discarding criteria shown in Figure 6, distance between each other (Figure 6c), similar grey intensity (Figure 6b) and similar size (Figure 6d), have been revealed enough to ensure that the two bright single regions belong to the same bubble. All of the segmentation tolerances are given by the user.

● Bubble outline:

After identifying all of the paired peaks and regions, the last algorithm step is to outline the bubbles. For the paired regions (regions pointed to by the magenta arrow in Figure 4g), the bubble to draw coincides with the smallest circumference that circumscribes the two regions (bubble pointed to by the magenta arrow in Figure 4h). For the paired peaks (regions pointed to by the yellow arrow in Figure 4f), a proportional law, empirically determined and involving the area of the region and the peak distance, has been implemented. Finally, all of the unmatched remaining regions are outlined as a whole bubble (small bubbles). At the end of the process, the algorithm removes the bubbles overlapped by other larger bubbles (bubble pointed to by the blue arrow in Figure 4f), provided the fact that they do not contribute to reliable information and, in most of the situations, comprise the conjunction of two adjacent bubbles (peak pointed to by the middle yellow arrow in Figure 4e). The final result for the example image is shown in Figure 4i, where an additional segmentation process is also displayed (blue regions), locating residual detected areas bubbles after collapse, bubbles out of the camera focused area in terms of field depth, incipient and vanishing bubbles, etc.

At the end of the recognition process and when all of the video frames have been processed, an output file with the results for each of the recognized bubbles X-center position, Y-center position, bubble characteristic length and current frame is generated.

#### 2.2.2. Side Projection

Similarly to the front projection, the analysis of the lateral footage returns information about the bubble’s instantaneous location on the side plane and the bubble characteristic side dimension. Again, a post-processing algorithm needed to be implemented to extract some information about the bubble size vs. life curve and its side projected trajectory. Obviously, there is no trustworthy possibility of locating nucleation sites, so no information about sites and frequencies can be obtained.

**Figure 7:**
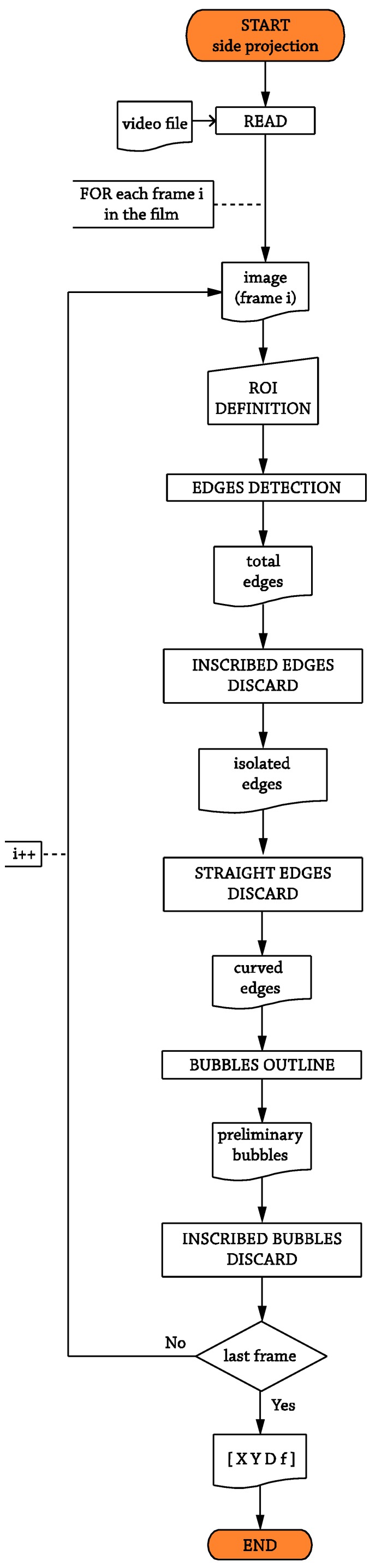
Flowchart for the side projection optical.

The lighting set for this projection comprises only one backlight used to illuminate the scene. The bubble optical pattern wanted here is to differentiate as best as possible the bubble silhouette from the background to perform a successful edge detection process. The basic operations carried out by the software to perform the optical recognition are sketched in the flowchart shown in Figure 7.

**Figure 8:**
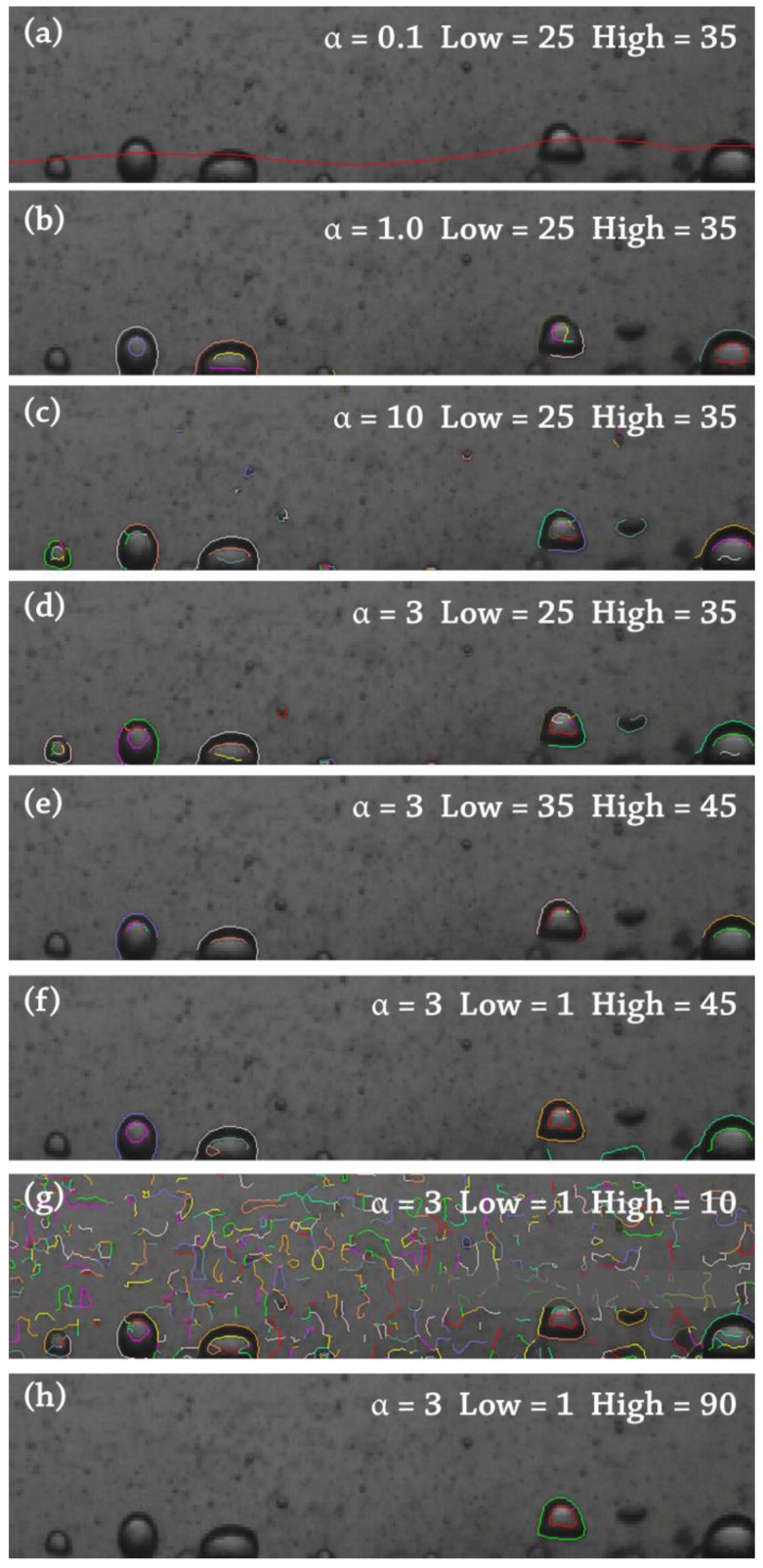
Filter value examples for the side projection.

Basically, apart from the aforementioned ROI definition, the side projection algorithm includes the following operations:

● Edges detection:

This operation detects the bubble candidate profiles employing an edge detector built-in function present in almost all of the commercial MV software. One of the first computational approaches to edge detection was proposed by Canny [52] and modified by Deriche [53] in the 1980s. Despite being developed almost thirty years ago, the Canny–Deriche approach is still widely used in edge detection applications, and many newer algorithms are somehow based on it. For this work, Deriche’s approach has been used since it gave better and slightly faster results. The classic implementation of the operator has three inputs apart from the image whose edges are going to be recognized, and the behavior for different values of the parameters is shown in Figure 8. The first parameter is the filter parameter α, which sets the desired level of localization when determining the bubble edge points. Low values for α yield very diffuse edges (Figure 8a), whereas higher values (Figure 8c) can result in counter-productive or simply non-productive results, depending on the level of noise in the image. Once the bubble edge points are determined, the function performs a hysteresis threshold fully described in [54] in order to link the points to edges. The points with an amplitude larger than “high” are immediately accepted as belonging to an edge, while points with an amplitude smaller than “low” are rejected, where “high” and “low” are the other two parameters. The remainder points are accepted as edges if they are connected to accepted edge points. Higher values for the “high” limit return few recognized edges with too much important information missed (Figure 8h). On the contrary, if the parameter “high” is set to a very low value (Figure 8g), the hysteresis threshold becomes very sensitive to small bubbles, dots and background non-uniformities. Once the “high” value is set, the between-limits range is determined by the “low” value. Variations of ‘low’ result in changes in the detected edges’ length, as the lower the “low” limit, the higher the amount of between-limits points recognized as edge points (Figure 8e,f). These parameters are decisive, not only in the success of the recognition operation, but also in the computing time, wasted in the case of having too many edges corresponding to useless areas of the image. Therefore, a good choice has to be made before the final analysis begins.

**Figure 9:**
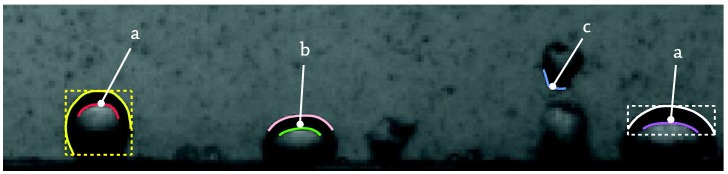
Discarding criteria in side projection: (**a**) inscribed discarded edges; (**b**) bubble due to the green edge will be outlined, but rejected in the final discarding process; (**c**) straight discarded edge.

● Edge discarding:

After the detection of bubble-candidate edges, a discarding process is carried out in order to ensure that all of the final edges really belong to a bubble. In this projection, two criteria to throw away non-bubble edges were performed, edges inscribed by an outer one (Figure 9a) and straight edges (Figure 9c). The latter criterion consists of analyzing the circularity of the selected edge, and in the case that the circularity value falls below a user input value, the edge is discarded due to its straightness. The circularity (ζ) is defined in Equation (1), where S_b_ stands for the area enclosed by the edge assumed to be a closed contour, and L_max_, is the distance from the centroid of the enclosed region to the furthest pixel belonging to the current edge.

(1) $$\xi =\frac{S_{b}}{\pi \;L_{\max}^{2}}$$

● Bubbles’ outline:

As well as in the front projection, at the final stage of the algorithm, the bubbles defined by the non-discarded edges are outlined, and an output file is written with the results X-center bubble position, Y-center bubble position, bubble diameter and current frame. Nevertheless, since it is possible depending on the parameter values assumed for the previous edge detection process to outline bubbles inscribed in an outer one mainly due to the edge detected between the center pale zone and the darker silhouette (Figure 9b), a final discarding phase has been performed to reject inscribed bubbles among the outlined candidate bubbles.

#### 2.2.3. Post-Processing

The files generated by the previous recognition methods are ready to be post-processed. In the case of the front projection, this operation computes the number of bubbles per frame, the instantaneous active site density and tracks all of the recognized bubbles in terms of diameter and position from where they appear until they collapse. The tracking method, which is how a bubble is considered a new or an inherited bubble, is briefly described here. Further information is given in [49].

It is clear that not all of the identified bubbles, frame by frame, represent a nucleation site. The tracking process commences with the analysis of the second frame. The first operation is to determine all of the new bubbles that have appeared precisely in this second frame comparing it with the previous one. To mark each of the 2nd frame bubble as an inherited or new bubble, a seeking area in the surroundings of the bubble is defined in the previous frame. In the case of the existence of a previous bubble, the bubble will be labelled as “past bubble”, since there is no possibility to know where the bubble has been really nucleated. On the other hand, the bubble is stored as a new bubble and labelled as “parent bubble”. After classifying all of the bubbles present in the 2nd frame, the process will continue with the following frames. For the subsequent frames (i frame), the classification is increased with to new types. The first new type is named as the “inherited bubble”, for those bubbles that derive from a “parent” one. The second new kind of bubble concerns bubbles in the previous frame (i-1), and applies to bubbles that have no continuation in the current frame; thus, they are labelled as “collapsing bubbles”. To improve the robustness of the process, after processing the entire footage, a completion operation is performed. For all of the bubbles recognized as “parent” or “collapsing”, the post-processing algorithm tries to complete the bubble life by looking some information up in the available stored data, the result of the aforementioned low-pass threshold operation.

In the application by the authors, the seeking area parameters consist of a minimum value, a user input value, according to the process uncertainty modified by a factor depending on the previously-computed velocity and acceleration, if any, of the centroid. The estimation of the uncertainty has to be done for each of the analyses individually. The uncertainty value should include the propagation of errors due to: equipment tolerances, sample size, recording speed and the detection method [47], taking into account the actual illuminance level, threshold values, etc., especially when distances and bubble dimensions are the magnitudes to be determined.

**Figure 10:**
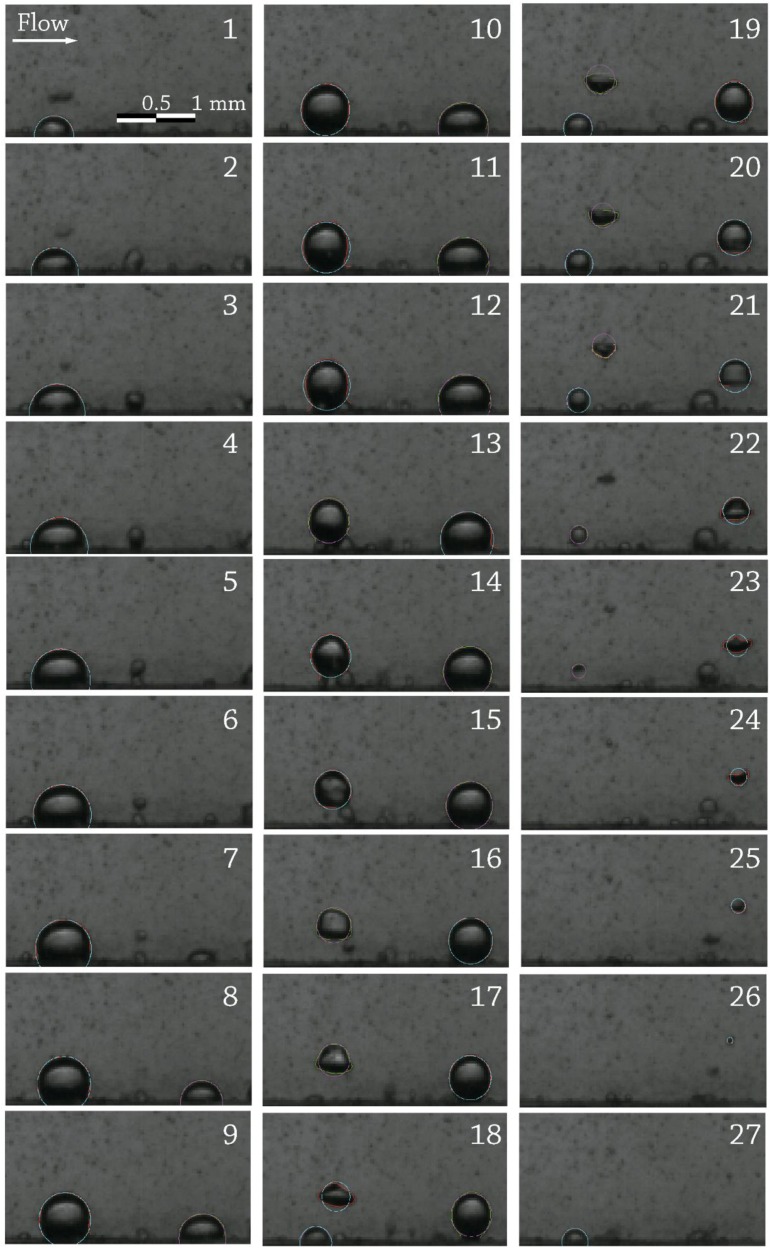
Side projection post-processing result example, T_b_ = 95 °C, p = 150 kPa, G = 241 kg/m^2^/s, v = 0.25 m/s, q_w_ = 300 kW/m^2^. Graphical output for recognized bubble contours (time interval between frames: 0.25 ms).

**Figure 11:**
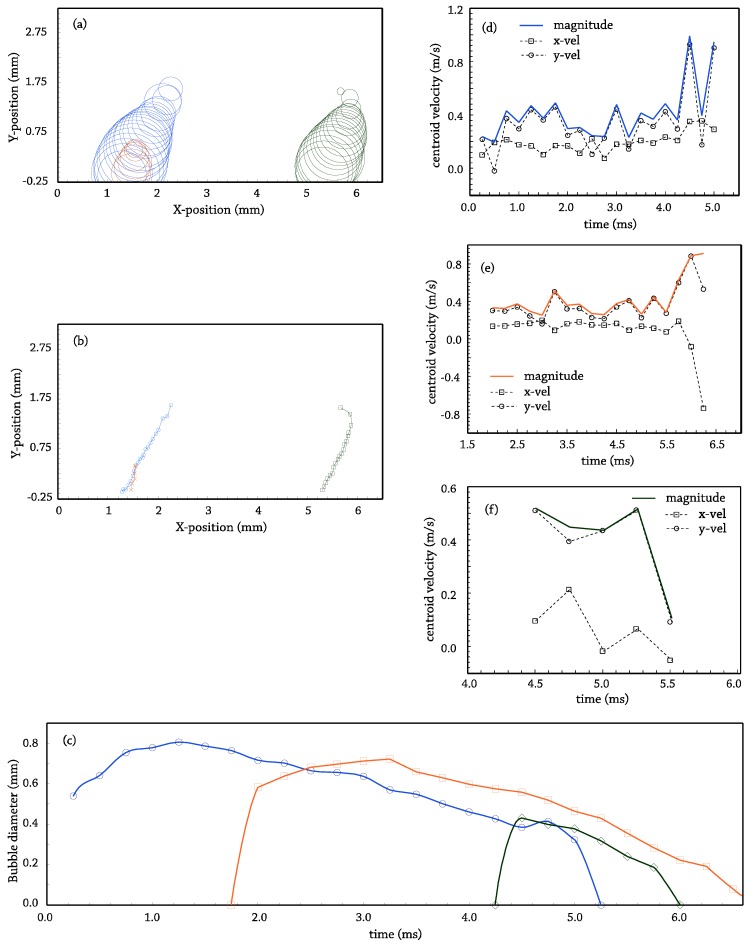
Side projection post-processing example for the frame sequence in Figure 10, T_b_ = 95 °C, p = 150 kPa, G = 241 kg/s·m^2^, v = 0.25 m/s, q_w_ = 300 kW/m^2^. (**a**) X-Y-size for detected bubbles; (**b**) bubbles’ centroid trajectories; (**c**) diameter-life diagram for recognized bubbles; (**d**–**f**) discrete calculation for the velocities of each of the recognized bubbles.

After bubble data are post-processed, any result in terms of the available data bubble position vs. time, bubble diameter vs. time, nucleation site density, releasing frequency and size for the bubbles of a site, trajectories and velocities of the bubbles, etc., can be extracted, depending on what the researcher is looking for. As an example, a graphical output of the application in nucleated boiling for the side projection is shown in Figure 10 (recognized bubble contours) and Figure 11 (post-processing output for the frame sequence).

## 3. Results and Discussion

This section discusses the algorithm’s results. To carry out the validation, two methods are proposed. First, to check the reliability in the bubble counting and recognition process, a human-eye versus machine vision comparison has been performed. Afterwards, the post-processed data in terms of bubble diameters and nucleation site density were benchmarked against other researchers’ experimental data.

To compare the MV output and the human-eye, two tests have been carried out. Firstly, two sets of three workers have been selected. The first group was comprised of people who are totally familiarized with boiling images, the expert group, and another group was formed by people who had never worked in bubble counting and boiling image analysis, the novice group. The sample, which was the same for each observer, was comprised of a total of five images at representative working point frames recorded at T_b_ = 85 °C, p = 150 kPa, G = 484 kg/s·m^2^, q_w_ = 500 kW/m^2^. The results are shown in Table 2.

**Figure 12:**
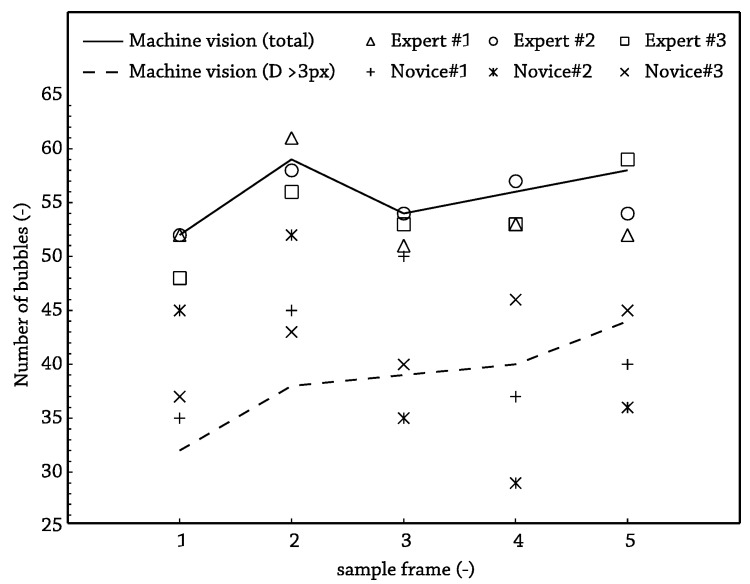
Detected bubbles by frame of the validation sample.

As expected, the first conclusion of this study was the relatively high deviation for the non-expert people in contrast to the expert ones: 6.1 bubbles versus 2.4 bubbles for the average standard deviations. A meaningful result is that the difference, frame-by-frame, between the averaged value for the total number of detected bubbles by the three experts and the output by the MV algorithm was always less than the standard deviation in the experts’ output, and for the worst case, only 5% of the average bubble number was unnoticed. Another fact to reinforce the reliability of the machine recognition is that values for human experts equaled or sandwiched the algorithm’s output, as can be seen in Figure 12, where the dynamic threshold value was picked as the average of the selected values by the expert group. In the figure, it also becomes clear that the main difficulties for the inexperienced operators lay in the very small, less than three pixel in diameter bubbles, as only 15% of “big” bubbles were rejected by this group of validators.

**Figure 13:**
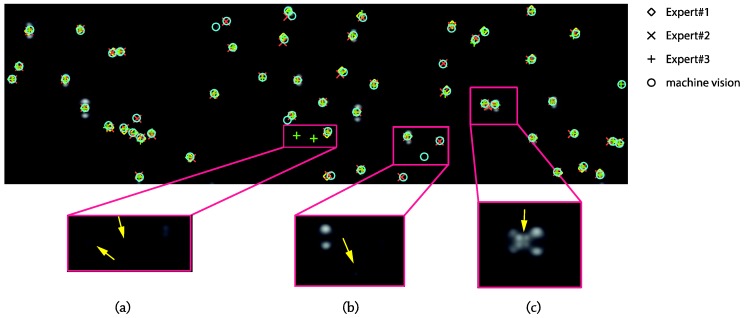
Discrepancies between human-eye and machine vision (MV) output. (**a**) Isolated bubble, rejected by MV but accepted by human-eye (**b**) Isolated bubble, accepted by MV but rejected by human-eye (**c**) merging bubbles.

The differences among the expert group members and between humans and MV output concern two aspects. On the one hand, when two bubbles are merging or arising from two close nucleation sites, the intersection area remains indefinite, and it is subjective to consider it a middle bubble or not. In fact, as can be seen in Figure 13 and in the zoomed region (c), only one expert operator has identified the two bright dots in the middle. The algorithm in this case has been programmed to reject the middle bubble if its center belongs two one of the other two side bubbles, which is the case. On the other hand, there are not very bright areas, which are difficult to decide if they are bubbles or not. In Figure 13a, two bright areas detected as bubbles only by Expert#3 have been zoomed; meanwhile, the arrowed zone in Figure 13b was identified as a bubble by the MV algorithm. The human brain is sensitive to possible optical illusions and surely makes decisions influenced by the environment, that is the threshold of the grey value for being considered or not as a bubble may change, whereas the algorithm establishes always an exact value. In order to improve the post-processing tracking detection in such situations, the optical recognition is also record, and these confused areas are stored (vapor residual areas; blue regions in Figure 4i), allowing the post-processing algorithm to select them as definitive bubbles if the continuity both backward and forward with other certain bubble regions is checked.

In terms of analysis time, the results have pointed out that the MV is more or less a thousand times faster than the human process, apart from other non-quantified human inherent factors, such as visual fatigue or non-repeatability.

**Figure 14:**
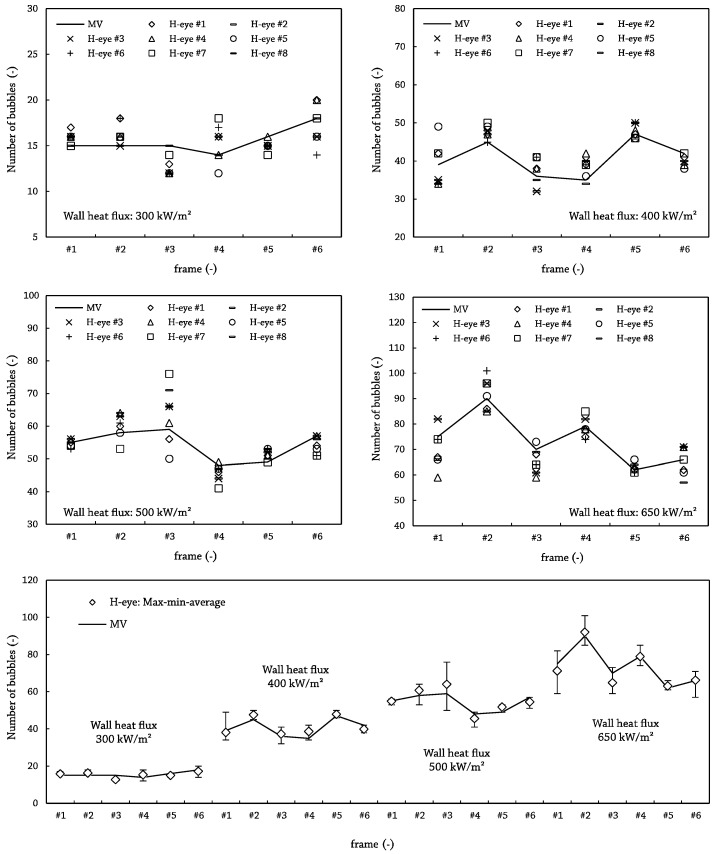
Validation results MV vs. human-eye for selected frames covering the full range of wall heat flux. Bottom: maximum, minimum and average value for all of the human observations and MV output. Top and middle: human single observations and MV output for the four selected values for the heat flux.

Once the differences between the expert group and the novice group were revealed, a second validation test has been made. This latter comparison included only the expertise group and has been completed covering all of the range in terms of wall heat flux. Thus, four samples comprised of six frames for each of the selected fluxes have been analyzed. After proving the statistical significance among all of the observations, the MV output lay again within the range output by humans, as can be seen on Figure 14. Furthermore, in 70% of the cases, the MV output was within the human average ± one standard deviation and, for the worst case, was nearer to two-times the standard deviation. Because of these results, the recognition algorithm demonstrates that it statistically gives the same results as human observers.

**Figure 15:**
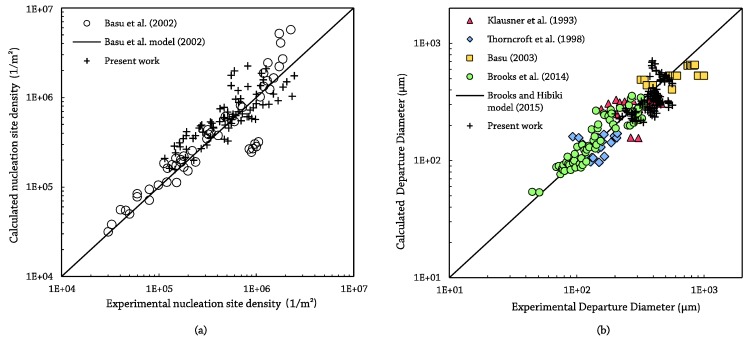
Validation results: MV output against data from other researchers: (**a**) nucleation site density; (**b**) bubble diameter.

Finally, in order to check the validity of the output, the obtained results have been post-processed in terms of bubble diameter and nucleation site density and then compared against other researchers’ data. To evaluate the performance in the characterization of nucleation site density, the results of Basu et al. [55] have been selected. The operational range for flow and thermal conditions of the present work is covered by the aforementioned dataset. Results for the nucleation density are shown in Figure 15a. Experimental points from Basu et al. comprise only data obtained for tests over a flat plate and for static contact angles from 75°–90°, similar surface conditions to those used in the present work. In addition, the output of Basu et al.’s proposed model has been also plotted. The present work’s data for the nucleation density present a root mean square percent error of 38.6%, within the ± 40% error bars declared in the original paper.

For the bubble diameter, data from Klausner et al. [56], Thorncroft et al. [25], Basu [57] and Brooks et al. [58] have been considered (Figure 15b). Moreover, the recent model from Brooks and Hibiki [59] to determine the bubble size has been plotted together with experimental data. The Brooks and Hibiki model predict present work data with average error of 21%, lower than the 22% reported by the authors for the previous dataset.

## 4. Conclusions

In this paper are presented two valid solutions for the analysis of high-speed video recording of the bubbling process encountered in subcooled boiling conditions at moderate high flux. Machine vision has been revealed as a valid method to outline and locate bubbles over the heated plate. On one side, small computation times allowing massive process, parallelization and batching options are among the big advantages in the application of machine vision systems to bubbling recognition, allowing sample sizes that under a manual process would be unmanageable. On the other side, the illumination setup process, the parameters’ adjustment and programming are the most remarkable challenges, but once the system is calibrated, machine vision has been found to be a very reliable and valuable ally in the bubble recognition process. Moreover, the proposed algorithms have been successfully employed with available boiling image sequences, taken from experimental facilities whose main goal was other than machine vision, as long as a minimum of precautions is taken.

## Figures and Tables

**Table 1:** Characteristics of the machine vision system.

Specification	Value
Resolution at max. speed	853 × 480 @ 10,000 fps
Image sensor	1696 × 1710 pixel with 8-bit dynamic range, monochrome
Sensor size	8-μm pixel size/13.6 mm × 13.7 mm @ 1696 × 1710 pixel
Light sensitivity	ISO 2200
Exposure time	from 1 μs to the inverse of the framing rate
Data interface	Gigabit Ethernet with RJ45
Maximum lamp luminous flux	up to 7700 lumens (dimmable)
Maximum lamp power	80 W

**Table 2:** Detected bubbles. Results for the two sets of human validators.

Frame	Algorithm	Expert#1	Expert#2	Expert#3	Novice#1	Novice#2	Novice#3
1	52	52	52	48	35	45	37
2	59	61	58	56	45	52	43
3	54	51	54	53	50	35	40
4	56	53	57	53	37	29	46
5	58	52	54	59	40	36	45
Total bubbles	279	269	275	269	207	197	211
Average time per bubble (s)	0.001 ^1^	1.04	0.96	1.25	1.06	1.35	1.06

^1^ The machine vision algorithm was run on an i7-3770 processor (Intel^®^, Santa Clara, CA, USA).

## Nomenclature

**Symbol**	**Designation**
G	mass flux (kg/s·m²)
p	pressure (Pa)
q	heat flux (W/m²)
T	temperature (K)
v	velocity (m/s)
	
**Subscripts**	
b	bulk conditions
w	wall conditions
	
**Abbreviation**	
CHF	critical heat flux
MV	machine vision
